# Cultural logics: a key issue in a Kaizen approach for malaria elimination

**DOI:** 10.1186/1475-2875-11-S1-P44

**Published:** 2012-10-15

**Authors:** Susanna Hausmann-Muela, Koen Peeters Grietens, Joan Muela Ribera

**Affiliations:** 1 Partners for Applied Social Sciences, PASS International, Tessenderlo, Belgium; 2 Swiss Tropical & Public Health Institute, Basel, Switzerland; 3 Institute of Tropical Medicine, Department of Public Health, Antwerp, Belgium; 4 Universitat Rovira i Virgili, Anthropology Department, Tarragona, Spain

## Background

Patients’adherence to malaria treatment is a key issue in malaria control and elimination. But adherence remains problematic even when drug supply is good, putting human behaviour at the forefront of interest. The current approach to behaviour change strongly relies on communication interventions [1]. Such information and communication for behaviour change addresses people’s *explicit* and *individual* knowledge, on the premise that deficient or incorrect medical knowledge can be corrected and change behaviour. It hardly considers people’s *implicit* and *collective* knowledge or *cultural logics*that underlie concepts and practices.

In this presentation, we propose a different approach. To use a metaphor, instead of observing, counting and measuring mushrooms, we study the mushroom’s *mycelium* - the invisible web of threads beneath the soil that acts in symbiosis with its environment and pushes the mushrooms, the only visible part of the entire organism, across the soil. To know about the mushrooms (actions), one needs to understand the *mycelium* in its environment (the ‘web of meanings’, in Geertz’ [2] terms).

## Cultural logics in malaria studies

Based on our work from Peru and Tanzania, we show how such cultural logics can explain certain behaviours. Concretely, we analyse the cultural logics behind malaria treatment adherence and the cultural construction of side effects in the Peruvian Amazon based on the hot-cold theory that permeates popular models of health and illness all over Latin America. We revise the cultural logic of witchcraft behind treatment-seeking behaviour and behind perception of symptoms in Tanzania. We look at the logic of illness progression to explain the observed sequence of treatment actions and we hypothesise about the logic of purity and danger for explaining apparently irrational behaviour for severe malaria, again in Tanzania.

## Kaizen approach for elimination

At first sight, the study of cultural logics might seem to provide interesting adds-on in understanding behaviour whose results can be included in the information and communication strategies for behaviour change. A closer look, however, paints a profoundly different picture. Cultural logics, i.d. the *mycelium* of knowledge, are implicit in people’s narrations, and they require anthropological skills and theories to identify them and make them explicit. The methodological approach is based on grounded theory and mixed methods. Grounded theory is an iterative process that entails inductive coding from the data. The analysis is done by “weaving in theoretical ideas and concepts without permitting them to drive or constrain the study’s emergent findings” [3]. The emphasis lies on ethnographic, qualitative (QUAL) in combination with a quantitative strand (quan) to test the relevance of identified cultural logics for practices.

The collective nature of cultural logics requires a particular approach for implementation. Information and communication that targets individuals for changing behaviour is unlikely to show an effect other than the mere accumulation of knowledge. Understanding cultural logics provides the clues for problem recognition at the collective level. But these logics moreover provide the tools for solving the problems. Like in the Kaizen approach [4], an intervention that takes cultural logics into account needs to be process- and people-oriented. Kaizen methodology includes making changes and monitoring results, then adjusting. Through continuous and incremental improvement, in dialogue with communities, the *mycelium* can slowly be reshaped and better mushrooms can grow.
